# Promotion and implementation effectiveness of World Health Organization's Caregiver Skills Training program in Taiwan

**DOI:** 10.3389/fpsyt.2022.904380

**Published:** 2022-08-31

**Authors:** Guan-Jye Seng, Yen-Nan Chiu, Wen-Che Tsai, Hsiang-Yuan Lin, Su-Chen Li, Mei-Ni Hsiao, Tseng-Jung Liu, Heng-Man Chen, Andy Shih, Ya-Chih Chang, WHO CST Team, Wei-Tsuen Soong

**Affiliations:** ^1^Department of Psychiatry, National Taiwan University Hospital, Taipei, Taiwan; ^2^Graduate Institute of Brain and Mind Sciences, College of Medicine, National Taiwan University, Taipei, Taiwan; ^3^Department of Psychiatry, College of Medicine, National Taiwan University, Taipei, Taiwan; ^4^Azrieli Adult Neurodevelopmental Centre, Centre for Addiction and Mental Health, Department of Psychiatry, University of Toronto, Toronto, ON, Canada; ^5^School of Occupational Therapy, College of Medicine, National Taiwan University, Taipei, Taiwan; ^6^Foundation for Autistic Children and Adults in Taiwan, Taipei, Taiwan; ^7^Department of Special Education, National Taipei University of Education, Taipei, Taiwan; ^8^Autism Speaks, New York City, NY, United States; ^9^Department of Special Education and Counseling, California State University, Los Angeles, CA, United States; ^10^Department of Mental Health and Substance Use, World Health Organization, Geneva, Switzerland

**Keywords:** autism spectrum disorder, developmental delays, World Health Organization Caregiver Skills Training, promotion, effectiveness

## Abstract

The World Health Organization (WHO) developed the Caregiver Skills Training for Families of Children with Developmental Delays and Disabilities (CST) with support from Autism Speaks to address the resource gaps and worldwide needs for interventions for children with developmental disorders or delays, especially those with autism spectrum disorder (ASD), and their families. Evidence has indicated that parent-mediated interventions benefit both caregivers and children by strengthening caregivers' knowledge and confidence and children's social communication skills and behavioral regulation. The CST-Taiwan team began the prepilot field trial in 2017 and developed the project to serve families in various locations. This study (1) delineated the adaptations and promotion of CST-Taiwan; (2) determined the program's effectiveness in the promotional stage, in terms of caregiver and child outcomes, and (3) examined the maintenance of its effects. The materials, delivery, and facilitator training procedure of the original CST were adapted to Taiwan. The quantitative data indicated that CST-Taiwan is a promising program, it positively affected caregiver knowledge and confidence and reduced the severity of the children's autistic symptoms. The 3-month follow-up results suggested that the effects persisted. Thus, CST-Taiwan, and its promotional strategies are feasible and effective.

## Introduction

Neurodevelopmental disorders are characterized by early childhood onset, alterations in central nervous system development, and functional impairments. Autism spectrum disorder (ASD) is a neurodevelopmental disorder characterized by persistent deficits in social communication and interaction and restricted and repetitive behaviors and interests (1). The prevalence is 1%−2% globally (2, 3) and 1% in Taiwan according to a nationally representative sample (4). The increasing prevalence (5) has placed a considerable financial and psychosocial burden on individuals, families and the society (6–8).

Early intervention is beneficial for autistic children in various regards, including social functioning, adaptive behaviors and behavioral regulation (9–11). Early intervention for developmental delays is highly demanded in Taiwan, with approximately 20,000 (1.5%) children younger than 6 years of age requiring such services every year (16,584 children in 2017, 19,103 children in 2018, 19,407 children in 2019, and 19,723 children in 2020) (12). In Taiwan, most early interventions are implemented in medical institutions in National Health Insurance system and nursery institutions in the social welfare system (13). Among early intervention services in Taiwan in 2020, 73% were implemented in medical institutions whose health care services are solely covered by the National Health Insurance, and 15.6% were implemented in nursery institutions (12). However, Taiwan's National Health Insurance only allows early interventions to be reimbursed as a non-intensive therapy (i.e., only one 30-min therapy session can be charged per person/week/institution). As such, daycares and child development centers that provide intensive treatment and care cannot satisfy the demand for interventions (3.7% of early intervention services in 2020) (12). Families often receive early intervention services at multiple institutions to increase treatment hours for their children. This distinct phenomenon in Taiwan results in inconsistency among the interventions (i.e., goals, theoretical bases, treatment principles, and skills) and requires time for travel, thus imposing a considerable extra burden on families, especially those living in rural area with limited access to services (14).

Parent-mediated interventions may compensate for this problem. Such programs require two key components: providing parents with information regarding ASD and helping them develop parenting skills (15). Parents' involvement in interventions for ASD improves parent–child interaction, generalization of the skills to daily living, increases consistency of interventions chose by the families, and enables parents to receive psychosocial support (16–18). In addition, parents trained in interventions can continue employing strategies in their children's daily lives (19, 20), thus generalizing and maintaining the treatment effect (21). Moreover, parent-mediated interventions increase parents' confidence in caregiving, reduce parental stress, improve quality of life, and lead to better parent-child relationship (16, 21–23). Improvement in parent–child interaction is a potential mediator in reducing autistic symptoms (24). Meta-analyses have revealed that parent-mediated interventions significantly reduced clinical symptom severity of the children (e.g., small effect size on parent-reported communication skills and medium effect size on challenging behaviors) (16–18). Earlier empirical studies have also revealed effects on children's sociability (25) and emotional regulation (26).

The World Health Organization (WHO) developed the Caregiver Skills Training Program for Families of Children with Developmental Delays and Disabilities (originally Caregiver Skills Training Program for Families of Children with Developmental Disorders or Delays, CST) with support from Autism Speaks (AS) (27) to improve caregiver skills and facilitate caregiver–child interaction, according to the WHO's mhGAP Intervention Guide (28). The program is effective, freely available, deliverable by non-specialists, feasible with limited resource, and can be integrated into other services (29). The WHO CST is open to all caregivers (not limited to parents) of children with developmental disorders or delays. The CST program consists of nine caregiver group sessions with manualized guides, three home visits, and three telephone calls and is based on the principles of natural developmental behavioral interventions (NDBIs) (29). The program is delivered according to established manuals by facilitators who are trained through WHO CST training program (i.e., Training of Trainers, ToT). The group sessions cover specific topics, including engagement, building up routines, communication, behavioral regulations and management, learning new skills, and caregiver's well-being (29). The caregiver role-play during group sessions and at-home practices after group sessions are used to strengthen skills from each session. Home visits are conducted prior to, midway through, and at the end of the group sessions to engage, coach, and support families, set goals, and evaluate the progress. The telephone calls are implemented after group sessions to clarify the group's focus and offer support. Although the WHO CST program was designed for global use, the materials and delivery process should be adapted for each context and to suit local needs (29, 30) to make the program relevant and feasible for implementation. The process of evaluating the feasibility and accessibility of the WHO CST consists of three stages: adaptation, prepilot and pilot stages (29, 31). Ideally, the adaptations were made prior to and within the prepilot stage. The adaptations were tested in the pilot stage to ensure the program is feasible and acceptable for dissemination in the local context. The program had been adapted and implemented in both low-income countries and high-income countries (30–32). The previous studies indicated that the WHO CST program is both acceptable and relevant to the low-resource contexts (e.g., Ethopia and India) (30, 31) and to the high-resource context (e.g., Italy) (33). A pilot randomized controlled trial study in Italy reported the promising effectiveness on joint engagement of caregiver–child interaction, parent stress, parenting self-efficacy and child gestures (32). The Taiwan CST team began adaptation and field testing in 2016, completed these steps in early 2019, and advanced to promotion in 2019. This study explored (1) the adaptations of CST-Taiwan, (2) its promotion, and (3) its effectiveness during the promotion stage.

## Materials and methods

### Adaptation process

This study is a part of the CST Taiwan Adaptation and Implementation Project launched by the Department of Psychiatry at National Taiwan University Hospital (NTUH) and the Foundation for Autistic Children and Adults in Taiwan (FACT). The core local team, consisting clinicians at NTUH and workers at FACT, was established in 2016 which lead the work of translation of “WHO CST Field Test version 1.0”. The materials, namely the facilitator guide, participant booklet, home visit guide, consent forms for families, and the monitoring and evaluation framework, were translated into Traditional Chinese (WHO CST Traditional Chinese version 1). Four master trainers were trained by the WHO-AS CST team and delivered the prepilot groups in 2017 in two institutions (one medical institution and one social welfare institution) in Taipei City, a metropolis. Before the prepilot groups, some adaptations of CST-Taiwan were developed on the basis of preparation meetings and rehearsals, yielding the WHO CST Traditional Chinese version 2, which was used in the prepilot groups. Because the WHO CST team released the WHO CST Field Test version 2.06 in 2018, our new version, based on WHO CST Field Test version 2.06 and a review of the prepilot groups in Taiwan, were used in the pilot groups in 2018 (i.e., the WHO CST Traditional Chinese version 3). Minor adaptations were made on the basis of the pilot study, leading to the latest version used in the promotion, the WHO CST Traditional Chinese version 4. In 2017 and 2018 (i.e., prepilot and pilot stages), CST-Taiwan implemented six caregiver groups by 12 facilitators (including the four master trainers) in five institutions, three in Taipei city and two in Kaohsiung (a city in southern Taiwan). We recruited 31 families (caregivers: *n* = 31, mean age=39.4 years, standard deviation [SD] = 6.1; children: *n* = 31, mean age = 4.0 years, [SD] = 1.0] during the prepilot and pilot stages in 2017 and 2018. Local adaptations in Taiwan were submitted to the WHO CST team for approval. The qualitative data collected during the adaptation process will be reported separately.

### Training of trainers (ToT)

In 2017, WHO-AS CST trainers held a 5-day ToT in Taiwan. Four specialists (one clinical psychologist, two occupational therapists, and one early intervention teacher) participated in live practice with children and submitted the Adult-Child Interaction fidelity videos to a WHO-AS CST trainer for verification. The WHO-AS CST trainer supported the subsequent posttraining practices as a technical consultant. After completion of the Adult-Child Interaction fidelity verification and the prepilot groups, the four master trainers led the first ToT for facilitators in Taiwan in 2018 (i.e., the pilot stage) and collected feedback from the facilitators for further adaptation. Thereafter, eight facilitators delivered four CST groups (two facilitators jointly delivered one group in pair) in Taipei and Kaohsiung at the pilot stage under the full supervision of the master trainers to ensure the procedural fidelity and to collect information to verify the local adaptations. The WHO-AS CST consultant verified the fidelity videos. ToT required adaptation for Taiwan because of the master trainers' workload, geographic restrictions, and the need for facilitators for promotion. Adapted ToT in Taiwan were held annually to scale up the promotion, and 15 facilitators maximum were trained a year.

We started the promotion stage in 2019. New institutions were invited to participate, and the four master trainers trained new facilitators each year. We targeted two cities or counties each year. Ideally, we sought to invite one medical institution and one social welfare institution (e.g., preschool education and early intervention organizations) in each targeted city or county; this was because early intervention services are provided in various institutions in Taiwan, and thus, we sought for representativeness of institutions (13). In the new institutions, newly trained facilitators would hold one CST group under the master trainers' supervision. The group enrollment ranged from four to six families. Institutions and facilitators who had previously participated were invited to continue the program in the following years after accreditation.

A review meeting was held at the end of each year of promotion. The CST-Taiwan core team, master trainers, facilitators, the leaders of the institutions, and the WHO-AS CST consultants attended the meetings. The implementation process, feedbacks, and challenges were reviewed. Suggestions of revision for future use were discussed and finalized, after approval by the WHO-AS consultants.

### Enrollment of families

Information regarding the Taiwan CST program was shared on the FACT website and advertised by the participating institutions. The families registered by themselves with or without referrals. To participate, families were required to have (1) children aged 2–6 years with (2) clear evidence of developmental delays, such as a confirmed clinical diagnosis (ASD was prioritized), developmental evaluation reports, or a disability certification; (3) the caregivers' commitment to participate in the group sessions and home visits; and (4) a basic level of reading and spoken Mandarin Chinese. However, we provided a brief translation of the materials (i.e., the whole participant booklet was translated into Simplified Chinese and the Key Messages and the Tips were translated into Vietnamese) to help caregivers who unfamiliar with Traditional Chinese. Usually, the main caregivers are the parents of children with developmental delays. Nonetheless, aunts, uncles, or grandparents could be the caregivers for some families because of the distinct family dynamics. Recruitment required the collective agreement of the Taiwan core team and participating institutions. During promotion stage in 2019 and 2020, we recruited 91 families. The data collected during the promotion stage were used in the analysis of effectiveness. The demographic data of the participants consisted of the child's age, sex, and treated/non-treated prior to the CST (i.e., whether the child had been treated through any type of early interventions; dichotomous variable) and the caregiver's age, relation to the child, educational level, and ethnicity.

All of the procedures during the prepilot and pilot field trials were approved by NTUH Institutional Review Board (#201703123RIND). We obtained written informed consent from the parent or substitute decision maker of each participated child after explaining the present objectives and procedures.

### Measurements

#### WHO caregiver knowledge and skills test

The WHO Caregiver Knowledge and Skills Test is a 5-point caregiver-reported questionnaire developed by the WHO CST team (unpublished) and consists of 24 items measuring the caregivers' understanding of the key principles of CST (i.e., knowledge). The higher score represents a stronger understanding of WHO CST caregiving (31). The internal consistency was high in our sample who completed baseline, postintervention, and follow-up tests, indicating high reliability (Cronbach's α of baseline/postintervention/follow-up test was 0.78/0.78/0.80).

#### Caregiver self-efficacy questionnaire

The Caregiver Self-Efficacy Questionnaire (CSQ) is a caregiver-reported questionnaire developed by the WHO CST team (unpublished) and consists of 13 items scored from 0 to 5 points to measure caregivers' confidence in the strategies delivered by WHO CST. The internal consistency was high in our sample who completed baseline, postintervention, and follow-up tests, indicating high reliability of this measure (Cronbach's α of baseline/postintervention/follow-up test was 0.89/0.91/0.93).

#### Family empowerment scale

The Family Empowerment Scale (FES) is used to measure the family empowerment in caregiving for children with special needs. It is a 5-point caregiver-reported questionnaire consisting of 34 items (34). The internal consistency in our sample who completed baseline, postintervention, and follow-up tests was high, indicating high reliability (Cronbach's α of pre-/post-/follow up test was 0.91/0.94/0.94).

#### Autism treatment evaluation checklist

The Autism Treatment Evaluation Checklist (ATEC) is a caregiver-reported questionnaire suggested as a comprehensive tool to monitor the treatment effect and progress of children with ASD. As a caregiver-reported measure, it has been validated in comparison of professional-rated measures with a well-established validity (35, 36). Four subscales are used to assess child outcomes, namely speech/language/communication (14 items, 3-point scale), sociability (20 items, 3-point scale), sensory/cognitive awareness (18 items, 3-point scale) and health/physical behavior (25 items, 4-point scale). The scores for the speech/language/communication scale and sensory/cognitive awareness scales were reversed to match the other two scales. Higher scores represented more severe autistic symptoms. The internal consistency in our sample who completed baseline, postintervention, and follow-up tests, indicating high reliability of the Chinese version (Cronbach's α of baseline/postintervention/follow-up test was 0.94/0.94/0.94 in the first subscale; 0.92/0.94/0.90 in the second subscale; 0.91/0.93/0.92 in the third subscale; 0.84/0.85/0.82 in the fourth subscale).

### Quantitative data collection

The Caregiver Knowledge and Skills Test, CSQ, ATEC were also translated into Traditional Chinese by the CST-Taiwan team during the adaptation process, while the Chinese version of FES was translated and validated in 2011 (37). The participants completed all measurements before the intervention (i.e., the questionnaires were given to the caregivers at the first home visits and sent back to facilitators at the first group session, which was the baseline), immediately after the intervention (postintervention), and 3-months after the intervention (follow-up). For families with two caregivers who participated in all sessions, the two caregivers completed separate questionnaires for caregiver-related outcomes.

### Statistical analysis

The demographics of the participants and their children are presented descriptively. The missing value rate of the questionnaires was 0.2%. Multiple imputation was used to replace missing values (38, 39) if the missing values presented randomly. A repeated-measures analysis of covariance (ANCOVA) was performed to compare the baseline, postintervention, and follow-up data with adjusting for caregiver age and educational level on WHO Caregiver Knowledge and Skills Test, CSQ and FES scores, and child age, sex, and treatment history and caregiver age on ATEC. We used Tukey's test to conduct *post hoc* analysis for the variables with significant differences.

To address the concerns regarding differences between families with whom we did not follow up, which may influence the interpretation of the follow-up data, we compared the demographics, baseline of caregiver and child outcomes between these two groups. In addition, the facilitators delivering the CST had three experience levels (i.e., A: two experienced facilitators, B: one new facilitator and one experienced facilitator, and C: two new facilitators; detailed in Section Adaptations of training of trainers (ToT) and supervision). Thus, we preformed ANCOVA to compared the changes between baseline and postintervention among these groups and to justify the analyses in which the effects of the facilitators' experience level on effectiveness were not controlled for. The baseline scores and Baseline× Group interaction term were included as covariates in the ANCOVA (40). These analyses are presented in the Supplementary materials. Providing the exploratory nature of this study, the presented results were not corrected for multiple tests.

## Results

### Local adaptations of CST-Taiwan

Several local adaptations were developed from the experience with the prepilot and pilot field trials, focus groups with facilitators and caregivers, and the comments during the review meetings for the core local team. The adaptations are summarized in Table 1.

**Table 1:** Adaptation summary of CST-Taiwan.

	**Planning and translation (2016)**	**Prepilot stage (2017)**			**Pilot stage (2018)**		
	**Translation and adaptation**	**ToT**	**Prepilot groups**	**Adaptation (2018)**	**ToT**	**Pilot groups**	**Adaptation (2019)**
Purpose	To translate the WHO CST materials to Traditional Chinese for local use.	To investigate the feasibility and the acceptability of the translated/adapted materials and the delivery process. To identify barriers of local implementation which may need adaptations.			To test the feasibility and acceptability of the adaptations based on the prepilot stage. To make further adaptations for promotion stage.		
Personnel	The local core team and 8 child therapists at NTUH.	WHO-AS CST Trainers, the local core team and potential master trainers.	The local core team and 4 master trainers.	The local core team and 4 master trainers.	The local core team, 4 master trainers and 8 facilitators.	The local core team, 4 master trainers and 8 facilitators from 4 institutions.	The local core team and 4 master trainers.
Adapted materials						
Facilitator guide	●	●		●			●
Participant booklet	●	●		●			●
Home visit guide	●	●					
Consent forms	●						
Monitoring and evaluation framework	•						
Version of materials	WHO CST Field Test version 1.0 was translated to WHO CST Traditional Chinese version 1.	Used WHO CST Traditional Chinese version 1, further adapted to version 2 during the rehearsals.	Used WHO CST Traditional Chinese version 2.	Adapted to WHO CST Traditional Chinese version 3.	Used WHO CST Traditional Chinese version 3.	Used WHO CST Traditional Chinese version 3.	Adapted to WHO CST Traditional Chinese version 4.
Summary of adaptations	Translation and sociocultural adaptations.	Unifying the format of facilitator guide and participant booklet.		Updated the version to WHO CST Field Test 2.06; adding theoretical bases to facilitator guide; highlighting and reformatting.			Minor change of wordings to make the texts plain.
Adapted ToT							
Criteria of facilitators					●		
Materials for ToT		●		●			
Format		●			●		
Contents of training					●		
Summary of adaptations		Establishing the play kits; adding rehearsals.		Translated Adult–Child Interaction Fidelity Scale.	Licenses and experiences required for facilitator; ToT was divided into two 2.5-day sessions 2 weeks apart; adding introduction of theoretical bases; increasing hours for hands-on practice.		
Adapted CST delivery							
Enrollment of			●				
participants						
Delivery personnel						●	
Group sessions						●	
Telephonic sessions			●				●
Home visits							●
Summary of adaptations			Change the recruitment children's age; 3 telephone calls changed to 7 telephonic sessions.			Two facilitators (rather than one master trainer and one facilitator) joint-delivery under supervision of master trainers; using prerecorded demonstration.	Establishing recording forms for telephonic sessions and home visits.

ToT, training of trainers; NTUH, National Taiwan University Hospital; FACT, Foundation for Autistic Children and Adults in Taiwan.

The core local team of CST-Taiwan consists four child psychiatrists at NTUH (W-TS, Y-NC, W-CT, H-YL), and the chief executive officer at FACT(T-JL).

### Adaptations of WHO CST materials

Adaptations were made to the contents of the WHO CST facilitator guide and the participant booklet, namely linguistic and sociocultural adaptation and the formatting. The “characters” and “locations” in the stories and demonstration scripts were changed into local names; however, the illustrations were retained to depict families of numerous ethnic origins. The facilitator guide and the participant booklet were reformatted to make the Key Messages, Tips, and facilitation notes more recognizable to readers (e.g., highlighting passages).

### Adaptations of WHO CST delivery

The age range of recruited children was adapted to 2–6 years (2–9 years in the original WHO CST program). A major adaptation in the Taiwan CST delivery was the use of prerecorded videos as substitutes for live demonstrations. The two facilitators recorded the videos beforehand to ensure that they understood the strategies demonstrated. The number of telephone calls was increased from three in the original WHO CST program to seven (i.e., after every session without a home visit). Also, we added coaching and troubleshooting for any difficulties among home practice to the telephone calls which last at least 30 min. The caregivers were welcomed to send home practice videos for facilitators' review before telephone calls. Therefore, we renamed the telephone calls to telephonic sessions.

### Adaptations of training of trainers (ToT) and supervision

First, we adapted the selection criteria for the facilitators. Although the WHO CST was originally designed to be delivered by both specialists and nonspecialists, we established selection criteria to optimize the implementation of CST-Taiwan. Institutions recommended facilitators on basis of two criteria: (1) license in a field related to developmental early-intervention or education (e.g., certified therapists in medical institutions, teachers, and practitioners from early intervention organizations) and (2) at least 3 years of experience implementing any type of developmental early interventions.

Second, the training of facilitators, which originally included a 5-day ToT session and post-ToT practices under supervision, was also adapted. The 5-day ToT session was divided into two 2.5-day sessions with 2 weeks apart to suit the clinical workload and schedule and to have time for practice. We added a theoretical introduction to the CST to the facilitator guide to deepen the facilitators' understanding of the core principle. More videos were shared during the training course to illustrate the Key Messages and Tips of CST. The hands-on practice time with children was also increased. The facilitators were invited to use the Adult–Child Interaction Fidelity Scale (WHO CST team, unpublished) to offer feedback to each other, and master trainers provided debriefing sessions immediately after the hands-on practice time with children. The facilitators were required to send Adult-Child Interaction Fidelity videos to verify they meet the WHO CST requirements. We added 3 rehearsal sessions after the 5-day ToT session, prior to the implementation of CST groups. The newly trained facilitators rehearsed all sessions to familiarized themselves with the content and structure of the CST groups. For the post-ToT practices, we used two facilitators joint-delivery under supervision of master trainers, rather than the original model of one master trainer and one new facilitator joint-delivery, which is also an adaptation of WHO CST delivery. The master trainers demonstrated an example of the first home visit for the new facilitators. The facilitators subsequently implemented their first CST group in pairs under the supervision of master trainers. The facilitators were considered as fully trained after the fidelity verification, rehearsals, and post-ToT practices, consisting of observing the first home visit implemented by master trainers, and their implementation under supervision.

We established a hierarchical supervision model for the facilitators and institutions based on level of experience (i.e., A: online supervision as required for two experienced fully trained facilitators, B: partial supervision for one experienced fully trained facilitator and one new facilitator, and C: full supervision for two new facilitators) to ensure the fidelity of the CST delivery. We also created home visit and telephonic sessions recording forms according to the CST guidelines for home visits and telephonic sessions. The recording forms record information (e.g., the child's competencies, the concerns of challenges, goals and progress of home practice) collected through interview or live interaction with the child. The facilitators were required to complete the forms for master trainers' review.

### Promotion of CST-Taiwan

**Figure 1:**
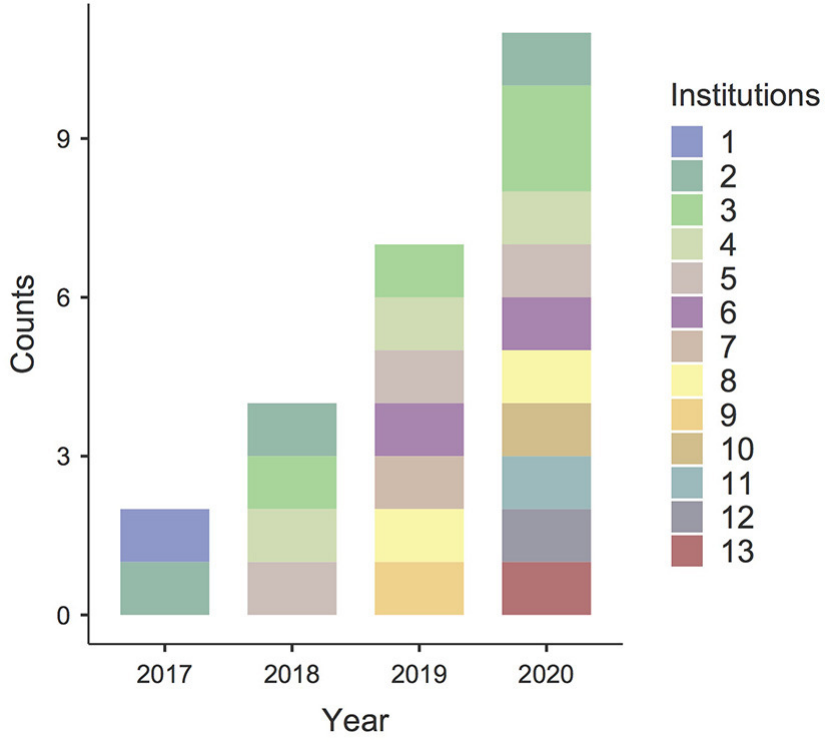
Promotion of CST-Taiwan. The number of institutions delivering CST increased over time. Two institutions implemented the first two groups in Taiwan (prepilot stage) in 2017. Four institutions implemented four groups (pilot stage) in 2018. During the promotional stage, seven institutions implemented seven groups in 2019. Ten institutions implemented 11 groups in 2020. By the end of 2020, 13 institutions had joined the CST-Taiwan program and implemented 24 groups.

**Figure 2:**
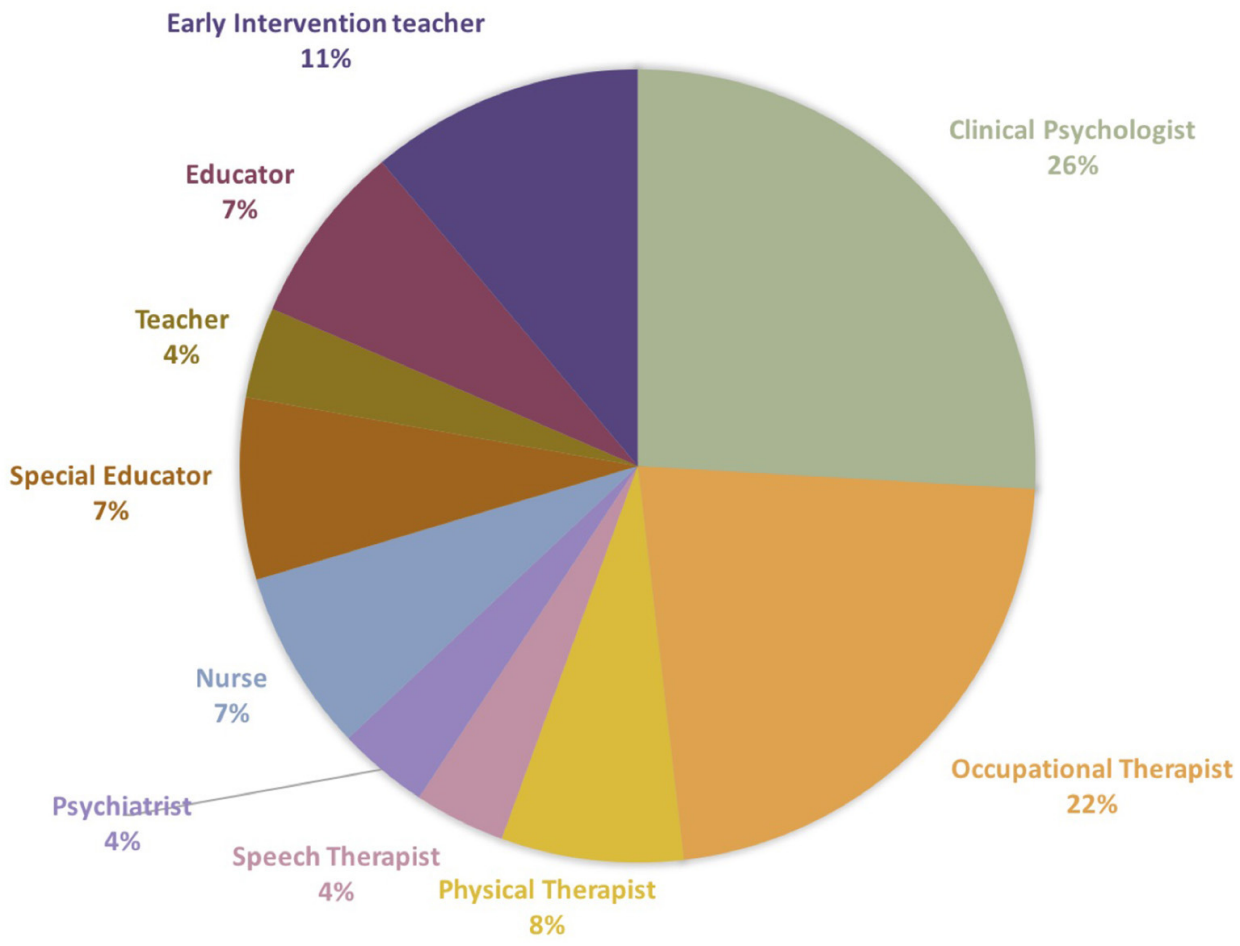
Types of the profession of facilitator at the promotional stage. The facilitators were clinical psychologists, occupational therapists, physiotherapists, speech therapists, special educators, educators, psychiatrists, early intervention teachers, and nurses.

During the study period (i.e., 2019–2020), 27 facilitators in 12 institutions implemented CST-Taiwan through 18 caregiver groups. The institutions were distributed across both urban and rural areas in Taiwan, including Taipei, Kaohsiung, Hsinchu, Yilan, Penghu, Hualien, and Taitung (Table 2 and Figure 1). A total of 33% of the facilitators continued to implement CST in the following years (included 5 facilitators, who were trained in 2018 and continued to implement CST in the following years). The facilitators were clinical psychologists, occupational therapists, physiotherapists, speech therapists, special educators, educators, psychiatrists, early intervention teachers, and nurses (Figure 2). Most facilitators reported a positive experience of implementing CST. Intensive supervision played a critical role in ensuring the new facilitators in correctly followed the CST guidelines, empowered them, and helped them resolve challenging problems in certain families. We used the quantitative data from the first 2 years of promotion (i.e., 2019–2020) to explore the effectiveness.

**Table 2:** Promotion summary of CST-Taiwan.

**Stage**	**Prepilot stage**	**Pilot stage**	**Promotion stage**	
Year	2017	2018	2019	2020
Regions (City/County)	Taipei	Taipei, Kaohsiung	Taipei, Kaohsiung, Hsinchu, Yilan, Penghu	Taipei, Kaohsiung, Hsinchu, Yilan, Hualien, Taitung
Number of newly trained facilitators	4 (Master trainers)	8	10	12
Number of implemented facilitators	4 (Master trainers)	8	14	21
Number of new institutions	2	3	4	4
Number of implemented institutions	2	4	7	10
Number of CST groups	2	4	7	11
Number of participating families	11	20	37	54

### Demographics of participants during promotion of CST-Taiwan

**Figure 3:**
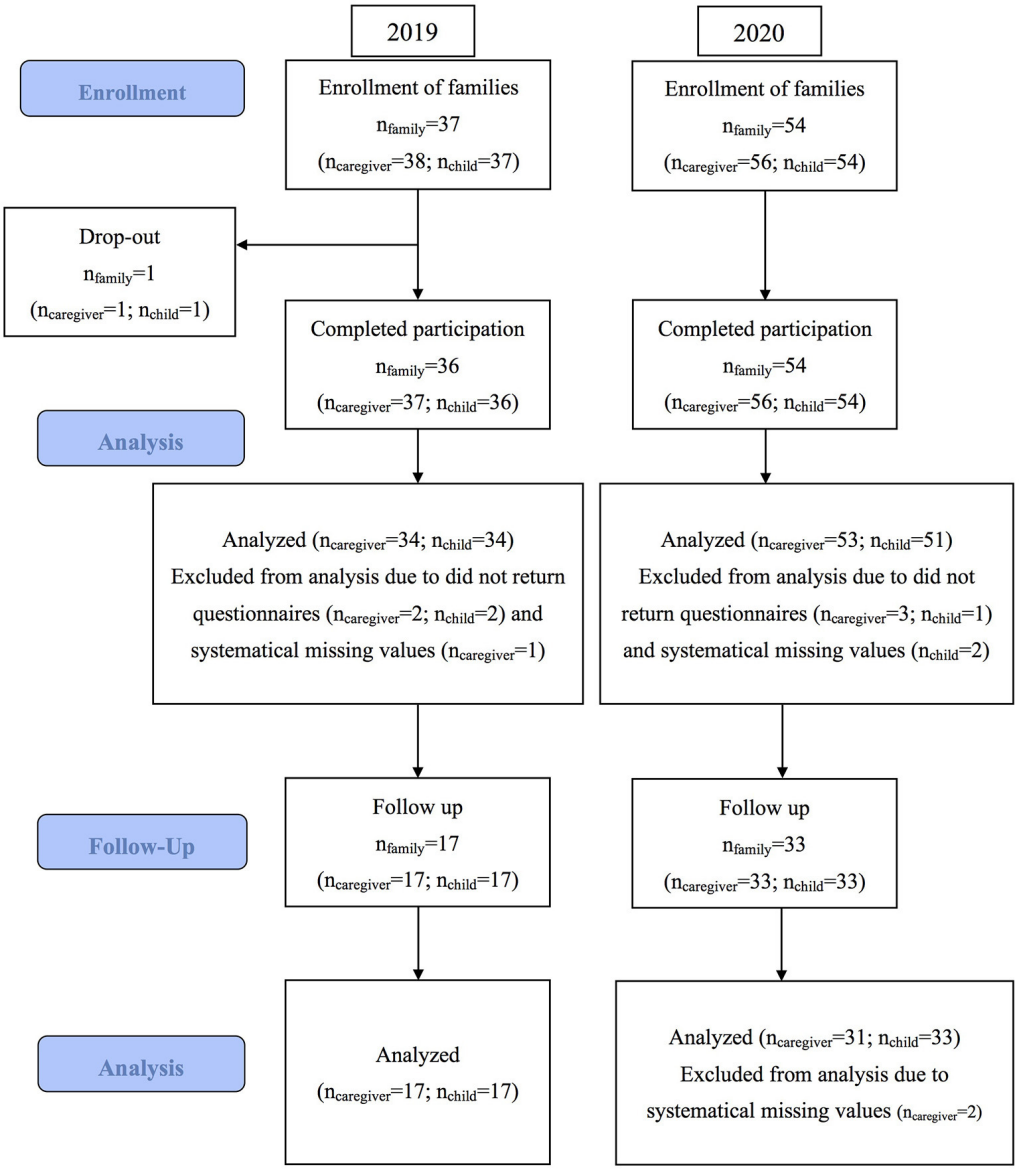
CONSORT flow diagram.

In 2019 and 2020, CST-Taiwan served 91 families (94 caregivers and 91 children, three families had two caregivers participating all sessions together). The mean age of the caregivers and children was 38.56 (SD = 5.81; range: 27–61) and 3.85 (SD = 1.07; range: 1.32–6.84) years, respectively. The caregivers were mostly the children's mothers (87.2%) and held a college degree or higher (81.9%). The baseline caregiver knowledge, confidence, and family empowerment were 96.06 (SD = 8.02), 38.76 (SD = 8.04), and 124.59 (SD = 14.9), respectively. Most of caregivers were Taiwanese (90 caregivers, including 3 indigenous), and four were foreigners. Most children were diagnosed as having ASD, but some were diagnosed as having developmental delays or disorders. Half of the children (51.6%) had been treated through any type of early interventions prior to CST. The mean baseline symptoms severity (total ATEC score) was 62.12 (SD = 25.3). Eighty-nine children in our sample were with a baseline ATEC score ≥20, a suggested minimal ATEC severity for using ATEC as a measurement (41). Only two children with ATEC total score lower than 20 (total score =18 and 19) at baseline, however, the clinical impression of ASD was apparent (Table 3). Only one family dropped out due to the busy schedule of the caregiver. Overall attendance rate was 91.7 % (Sessions 1–9: 95, 92, 95, 87, 91, 93, 91, 88, and 95%, respectively). Figure 3 presents a CONSORT flow diagram of the participants.

**Table 3:** Demographics of children and caregivers participating CST-Taiwan at the promotion stage.

**Children (*n =* 91)**	
Age, year [mean, (SD)]	3.85 (1.07)
Age range	1.32–6.48
**Sex [** ***n*** **, (%)]**	
Male	70 (76.9%)
Female	21 (23.1%)
Treatment history [*n*, (%)]	
Treated	47 (51.6%)
Non-treated	44 (48.4%)
Geographic areas [*n*, (%)]	
Rural	23 (25.3%)
Urban	68 (74.7%)
Baseline autistic symptoms severity^a^ [mean, (SD)]	62.12 (25.29)
Baseline autistic symptoms severity^a^ range	18–116.2
**Caregivers (*****n** =* **94)**	
Age, year [mean, (SD)]	38.56 (5.81)
Age range	27–61
Sex [*n*, (%)]	
Male	7 (7.4%)
Female	87 (92.6%)
Caregiver's relation to the child^b^ [*n*, (%)]	
Father	7 (7.4%)
Mother	82 (87.2%)
Grandparents	3 (3.2%)
Other	2 (2.1%)
Caregiver educational levels [*n*, (%)]	
Junior	3 (3.2%)
High	14 (14.9%)
College	55 (58.5%)
Graduate	22 (23.4%)
Ethnicity	
Taiwanese	87 (92.6%)
Indigenous	3 (3.2%)
Foreign	4 (4.3%)
Baseline caregiver knowledge^c^ [mean, (SD)]	96.06 (8.02)
Baseline caregiver confidence^c^ [mean, (SD)]	38.76 (8.04)
Baseline family empowerment^c^ [mean, (SD)]	124.59 (14.94)

SD, standard deviation.

a Baseline autistic symptoms were assessed by the total score of Autism Treatment Evaluation Checklist. Only 88 children had this data.

b Three families had two caregivers participating group sessions together.

c Only 92 caregivers completed these questionnaires, one father and one mother whose partner also participating groups session did not complete these questionnaires.

### Differences among baseline, postintervention and follow-up

A total of 87 WHO Caregiver Knowledge and Skills Test, CSQ and FES questionnaires were completed both at baseline and postintervention, and 48 were completed at follow-up; 85 baseline and postintervention ATECs were completed, and 50 follow-up checklists were completed. We observed no significant difference in demographics and baseline child and caregiver outcomes between participants with whom we followed up and those with whom we did not (Supplementary Table 1). No significant difference in demographics and baseline child and caregiver outcomes was observed among the groups with various facilitator experience levels, but more children had been previously treated in groups delivered by two new facilitators compared to the groups delivered by one new and one experienced facilitator (Supplementary Table 2). We observed no significant difference in caregiver (i.e., knowledge, confidence, and empowerment) or child outcomes (i.e., all ATEC subscales and the total score) among the groups with various facilitator experience levels (Supplementary Table 3).

### Caregiver outcomes

With controlling for caregiver age and educational level, significant difference was observed between baseline and postintervention knowledge [*F*_(1,84)_ = 4.78, *p* = 0.032, *n* = 87, Table 4A]. Significant effect of time on confidence [*F*_(2,90)_ =3.80, *p* = 0.026, *n* = 48] was observed among difference among baseline, postinvention and follow-up. No significant difference was observed between the postintervention and follow-up results in *post hoc* analysis (Table 4B).

Table 4Caregiver outcomes-baseline, postintervention and follow-up differences.

**Table:** 

**(A) Baseline and postintervention differences**					
	**Mean (SD)**		**Repeated measured ANCOVA§**		
	**1 Baseline (*****n** =* **87)**	**2 Postintervention (*****n** =* **87)**	**Statistic (F)**	***p*** **–value**	**Effect size (**η^2^**)**
Knowledge	95.94 (7.92)	102.83 (7.41)	4.78	0.032	0.006
Confidence	38.86 (8.08)	49.17 (6.75)	2.94	0.090	0.005
Empowerment	124.71 (15.25)	135.04 (15.87)	<0.001	0.992	<0.001

**Table:** 

**(B) Baseline, postintervention and follow-up differences**							
	**Mean (SD)**			**Repeated measured ANCOVA§**			***Post-Hoc*** **(***p*_Tukey_**)**
	**1 Baseline (*****n** =* **48)**	**2 Postintervention (*****n** =* **48)**	**3 Follow up (*****n** =* **48)**	**Statistic (F)**	***p-*** **value**	**Effect size (**η^2^**)**	
Knowledge	96.15 (8.28)	102.30 (7.69)	100.86 (8.52)	2.21	0.115	0.006	—
Confidence	39.42 (7.72)	49.28 (6.79)	49.09 (8.14)	3.80	0.026	0.015	1 < 2,3
Empowerment	123.78 (14.8)	136.04 (15.9)	134.04 (16.1)	0.52	0.595	0.001	—

SD, standard deviation; ANCOVA, analysis of covariance.

§ caregiver's age and educational level included as covariates.

### Child outcomes

With controlling for child's age, sex, treatment history and caregiver' age, significant differences between baseline and postintervention on speech/language/communication, sociability, health/physical behavior, and the total score were observed [*F*_(1,80)_ = 11.95, *p* < 0.001; *F*_(1,80)_ = 5.04, *p* = 0.028; *F*_(1,80)_ = 4.47, *p* = 0.038, *F*_(1,80)_ = 9.23, *p* = 0.003, Table 5A). Significant effect of time on speech/language/communication, sociability, and the total score were observed in differences among baseline, postintervention and follow-up [*F*_(1.84,82.70)_ = 7.82, *p* = 0.001; *F*_(2,90)_ = 7.60, *p* < 0.001; *F*_(2,90)_ = 3.64, *p* = 0.030]. In *post hoc* analysis, no significant difference was observed between the postintervention and follow-up results in speech/language/communication and the total score, while no significant difference was observed in the baseline–follow-up and postintervention–follow-up comparisons in sociability (Table 5B).

Table 5Child outcomes-baseline, postintervention and follow-up differences.

**Table:** 

**(A) Baseline and postintervention differences**					
	**Mean (SD)**		**Repeated measured ANCOVA§**		
	**1 Baseline (*****n** =* **85)**	**2 Postintervention (*****n** =* **85)**	**Statistic (F)**	***p*** **value**	**Effect size (**η^2^**)**
Speech/language/communication	13.20 (7.95)	10.95 (7.53)	11.95	0.001	0.002
Sociability	13.11 (6.63)	11.63 (7.43)	5.04	0.028	0.003
Sensory/cognitive awareness	15.50 (6.96)	13.49 (7.10)	2.94	0.090	0.001
Health/physical behaviors	20.54(10.93)	18.59 (10.68)	4.47	0.038	0.002
Total	62.34(25.12)	54.66 (25.15)	9.23	0.003	0.004

**Table:** 

**(B) Baseline, postintervention and follow-up differences**							
	**Mean (SD)**			**Repeated measured ANCOVA§**			***Post-Hoc*** **(***p*_Tukey_**)**
	**1 Baseline (** ***n** =* **50)**	**2 Postintervention (** ***n** =* **50)**	**3 Follow up (*****n** =* **50)**	**Statistic (F)**	***p*** **value**	**Effect size (**η^2^**)**	
Speech/language/communication	12.86 (7.84)	10.64 (7.44)	9.52 (7.74)	7.82	0.001	0.004	1 > 2,3
Sociability	12.48 (6.60)	11.40 (8.14)	10.90 (7.83)	7.60	<0.001	0.006	1 > 2
Sensory/cognitive awareness	15.66 (7.29)	13.98 (7.59)	12.48 (7.92)	0.34	0.711	<0.001	—
Health/physical behaviors	18.13 (9.11)	17.17 (9.08)	17.10 (8.57)	0.64	0.510	0.001	—
Total	59.15 (23.2)	53.19 (25.2)	49.99 (26.9)	3.64	0.030	0.003	1 > 2,3

SD, standard deviation; ANCOVA, analysis of ovariance.

§ child's age, sex, treatment history, and caregiver's age, included as covariates.

## Discussion

This study revealed that implementing the local adapted WHO CST is feasible in Taiwan. The content, delivery, criteria for facilitators selection, and ToT were adapted. The promotional model of CST-Taiwan expanded the service to cities and counties across rural and urban areas in Taiwan. The quantitative data indicated the preliminary effectiveness of CST-Taiwan in positively affecting the caregivers' knowledge and confidence and the children's autistic symptoms with a maintenance effect.

The CST was adapted in Taiwan to optimize implementation across the country. The content was adapted only slightly, suggesting that the WHO CST materials were already applicable to Taiwan. This is similar to the experiences from several WHO CST sites which suggested that the contents of WHO CST is feasible and acceptable for global contexts (30, 31, 33). In terms of delivery, the use of prerecorded videos and increased telephonic sessions strengthened the effects of the program on the caregivers' learning. As some demonstration scripts were complex for facilitators to act leading to a lower acceptability in caregivers (30, 33), prerecorded demonstration videos eased the preparation pressure of facilitators and increased the understandability of the caregivers. The facilitators emphasized and clarified key concepts by pausing or replaying the videos for the caregivers during the group sessions. The increased telephonic sessions provided one-on-one individual coaching after each group session, which is one of major adaptations of CST-Taiwan responding to the suggestions for intensifying the coaching in Salomone et al.'s work (33). The facilitators encouraged and coached the caregivers to do home practices through intensive follow-ups. When caregivers described challenges they faced during at-home practice between group sessions, the facilitators coached them and offered tips.

Although originally nonspecialists could implement CST, our facilitators were all specialists in developmental early interventions, which is a difference between CST-Taiwan and others (31). Because half of the children had received early interventions, the facilitators with experience in early intervention and related skills felt capable of answering caregivers' questions, and upholding the Adult-Child Interaction fidelity. In addition, CST groups were delivered by two newly trained facilitators in Taiwan, which is different from the WHO CST suggested that facilitators jointly deliver CST groups with master trainers (as a part of post-ToT practices). Thus, we used a different training format to provide more practice opportunities for facilitators. First, we introduced the theoretical bases of CST to the facilitators at the beginning of the ToT because not all were familiar with NDBIs. This benefited facilitators, who had various professional backgrounds, by aligning their professional perspectives with the concepts of NDBIs before learning CST; this may have prevented confusion and resistance among facilitators. This adaptation echoed Salomone et al.'s work which suggested a booster training module aimed at introducing NDBIs is needed (33). Second, ToT was adapted considerably to provide more opportunities for practice and to strengthen the facilitators' competencies. ToT was divided into two 2.5-day sessions 2 weeks apart; the facilitators were asked to practice what they had learned in the first session of the ToT during these 2 weeks. We also increased the opportunities for hands-on practice with delivering CST and facilitator–child interaction during ToT. In addition, we added three rehearsals before implementation. The unfamiliarity with the program materials may be a challenge for the facilitators when delivering CST (30). The added rehearsals were key to ensuring that the facilitators were well prepared to deliver CST to the families. The first rehearsal, prepared the facilitators for the first home visit and first three group sessions. In the second rehearsal, the facilitators rehearsed the second home visit and the fourth through sixth group sessions. The third rehearsal covered the last home visit and seventh through ninth group sessions. To ensure the facilitators delivered CST faithfully, the master trainers closely supervised the group sessions, home visits, and telephonic sessions. Established recording forms for home visits and telephonic sessions were essential for the master trainers to evaluate and provide feedback. Their supervision encouraged the new facilitators to strengthen their skills and correctly implement all CST procedures.

We adapted ToT on the basis of the learning framework, which involved three key components: knowledge, skills, and attitude (42). Comprehensive background knowledge of NDBIs (provided in the beginning of ToT) facilitated the learning of knowledge of CST. However, developing CST skills and changing attitudes toward early interventions are more challenging than acquiring knowledge. The observation and hands-on practices play a critical role in bridging the gap between “knowing what” and “knowing how” (43). The facilitators received more hands-on practices with children in ToT, observed the master trainers conducting the first home visit and discussed their home visits and telephonic sessions with the master trainers. These practices accelerated and facilitated the development of CST skills and strengthened the facilitators' competencies (44). Thus, our adaptations provided a feasible model to accelerate promotion without sacrificing quality.

CST-Taiwan adopted a progressive promotional model because of the limited numbers of master trainers who could supervise new facilitators. The maximum number of new institutions under simultaneous supervision was limited to eight to consider the workload of the four master trainers. The promotional strategy was to invite and train new facilitators each year to independently implement the CST in the following years of implementation, thereby increasing the number of institutions implementing CST. The one-third facilitators reimplemented CST in the following years, implying the high acceptance among facilitators and local feasibility (33). Most facilitators experienced growth in their professional fields and empowerment by helping the families with needs. The barriers to implementation were mostly practical. The WHO CST is provided to families free of charge, which creates barriers to promotion. Because the WHO CST entails high time and human resource costs, institutions often prefer chargeable services in the health-care system. The Taiwan CST core team asked that FACT subsidize experienced institutions (i.e., the facilitators who had completely led at least one CST group under supervision); however, the program was still not cost effective from the institutions' perspective. A future direction is to embed CST into National Health Insurance in Taiwan to provide CST to communities.

This study demonstrated the effectiveness of CST-Taiwan. The caregiver outcomes indicated the promise of this program. The caregivers learned the core concepts through the nine group sessions, three home visits and seven telephonic sessions. They also reported stronger confidence in caregiving. They felt capable of interacting with their children, facilitating their development, and coping with the stress of caregiving, which is consistent with previous studies (31, 32). This effect persisted for 3 months or more, indicating long-term changes in the caregivers' behaviors and attitude. The construction of WHO CST may have contributed to this promising learning effect. First, the Key Messages and Tips were presented repeatedly during group sessions through various ways including stories, illustrations, demonstrations, discussions and practice in pairs. Individuals are more likely to master knowledge and skills if information is presented in multiple ways (45). The WHO CST also strongly emphasizes the importance of integrating the strategies into daily home routines (29). The newly acquired strategies were immediately applicable to daily life and therefore strengthened the caregivers' knowledge and skills acquisition (46). The intensive one-on-one telephonic sessions after every group sessions, one of our adaptations, may also have contributed to the learning effect (25). The CST program may provide foundational training for families new to the early intervention system. In Taiwan, the accessibility of early intervention services may be higher than that in other countries because those in approved institutions are covered by National Health Insurance and government subsidies (13, 47). However, the search for services may lead to therapy shopping among parents (47), especially those in urban areas. For families who had participated in several interventions, CST may encourage families with children who previously received interventions to select evidence-based and effective interventions for their children (48). On the other hand, receiving CST also ease the caregivers' confusion of whether they are facilitating their child correctly, this is similar to the report from India addressing the positive experience toward CST program from the caregivers (31). The CST program can also complement the intervention service system by strengthening caregivers' skills. The improvement in caregiving knowledge and confidence persisted for all types of caregivers (in terms of age and education level). This indicates that CST can benefit caregivers of all backgrounds.

We observed that the severity of the children's symptoms of autism was significantly lower after the CST intervention, with adjustment for potential confounding factors (except for sensory/cognitive awareness). The change in communication, sociability, and the total score persisted for 3 months or more. The results suggest that CST reduced core autistic symptoms with consideration for potential confounders. The significant improvement of children's communication is also found in the pilot groups in India (31). To be noted, the improvement of children's gesture use in communication, which is a crucial component in CST, was reported in the randomized controlled trial study in Italy (32). However, there was no item assessing the gesture use in the communication subscale of ATEC, yielding a limitation of this study. The significant improvement in communication and sociability but not behaviors may have resulted from the order of the CST sessions and the measurement tool (i.e., ATEC). The sessions addressing communication and sociability were in the first half of the program. The caregivers started to learn and practice strategies to facilitate communication and sociability among the children earlier than they learned strategies for emotional behavioral regulation. The WHO CST sessions are arranged in a hierarchy; earlier sessions form the basis for the latter sessions, that is, the caregivers' role play and at-home practice during each session incorporate all strategies from prior sessions. Most caregivers set communication as their main goals at the first home visit. Because we provided individual sessions after each group sessions, the practice of skills to facilitate the children's communication was frequently addressed in telephonic sessions. Therefore, an accumulated effect of practicing these skills may have contributed to the significant improvement. The “dosage' of practicing behavioral regulation strategies may not have been sufficient to create a significant improvement at the end and follow-up time points. However, further examination to test this hypothesis is required. In addition, more realistic examples of strategies for challenging behaviors in the CST content had been called for (33), indicating that the needs of some caregivers for behavior regulation strategies were not fully covered in the current version of the CST materials. Behavioral outcomes are included in the physical/health/behaviors subscale in ATEC. The subscale consisted of not only items assessing behaviors but also items assessing toileting (not covered in CST), self-care and autistic rigidity. Thus, the nonsignificance of the changes in behavior may have been related to the measurement tool. Future research with different measurements of behavioral regulation should be considered. A longitudinal epidemiological study observed a significant reduction in ATEC scores over time (41, 49); the changes in the total score were large in younger children, who were the majority in this study. Thus, this study cannot rule out the effects of natural developmental factors on effectiveness because we did not have a control group to demonstrate that CST, rather than time, led to improvement. The characteristics of the children and caregivers determine the effectiveness of parent-mediated interventions (50). Additional mediation analyses with detailed characteristics information are required to determine relationships between effectiveness and associated factors.

The strength of this study is the establishment of a strong promotional model to ensure quality and expand the program. Although the results indicate the promise of CST-Taiwan, this study has several limitations. The main limitation of this nonexperimental study is the lack of a control group, which weakens the results. To match the original target population for the WHO CST, CST-Taiwan was open to families with children with developmental concerns. Therefore, a confirmed diagnosis or assessments were not necessarily required, which may have yielded unexpected confounders. Most caregivers had a high education level (the majority had a college degree or higher). Thus, the findings may not be generalizable to caregivers with low education levels. Additional child and caregiver characteristics should be considered in further research (e.g., types and lengths of treatments prior to CST, children's cognitive and language levels of the children, and families' socioeconomic status).

This study revealed that the adapted format of the WHO CST, namely CST-Taiwan, and its promotional model are feasible. CST-Taiwan has significantly improved caregivers' knowledge and confidence. It has also reduced the autistic symptoms among the children overall. The follow-up results suggested that the positive effects remained even after the program.

## Data availability statement

The datasets generated and analyzed during the current study are not publicly available due to the research ethics regulation in Taiwan. However, they are available from the corresponding author on reasonable request.

## Ethics statement

Ethical review and approval was not required for the study on human participants in accordance with the local legislation and institutional requirements. Written informed consent to participate in this study was provided by the participants' legal guardian/next of kin.

## Author contributions

W-TS led the team as the principal investigator of WHO CST Team: Taiwan Adaptation and Implementation Project. Y-NC, W-CT, and H-YL are the co-principal investigators of the project. S-CL, M-NH, G-JS. and H-MC are master trainers providing trainings for facilitators in Taiwan. T-JL contributed to administrative works including seeking for financial resource and connecting with the governmental agency. AS introduced WHO CST to Taiwan and attended preparatory and review meetings to provide comments for the development of CST-Taiwan. Y-CC contributed to technical supports including training of master trainers, verifying the fidelity of facilitators and master trainers, and approved all technical adaptations of CST-Taiwan. The WHO CST Team developed the CST materials and provided the monitoring and evaluation framework. G-JS and W-TS analyzed the data and wrote the manuscript with input from all authors. All authors contributed to the article and approved the submitted version.

## Funding

Open access funding provided by Foundation for Autistic Children and Adults in Taiwan. This study was supported by Social and Family Affairs Administration Ministry of Health and Welfare and Yong-Tai Chen Charitable Trust.

## Conflict of interest

Author AS was employed by Autism Speaks. The remaining authors declare that the research was conducted in the absence of any commercial or financial relationships that could be construed as a potential conflict of interest.

## Publisher's note

All claims expressed in this article are solely those of the authors and do not necessarily represent those of their affiliated organizations, or those of the publisher, the editors and the reviewers. Any product that may be evaluated in this article, or claim that may be made by its manufacturer, is not guaranteed or endorsed by the publisher.

## References

[1] American Psychiatric Association. Diagnostic and statistical manual of mental disorders (DSM-5^®^). (Washington, DC: American Psychiatric Pub) (2013).

[2] Zeidan J Fombonne E Scorah J Ibrahim A Durkin MS Saxena S. Global prevalence of autism: A systematic review update. Autism Res. (2022) 15:778–90. 10.1002/aur.2696 35238171 PMC9310578

[3] Lord C Charman T Havdahl A Carbone P Anagnostou E Boyd B. The Lancet Commission on the future of care and clinical research in autism. Lancet. (2022) 399:271–334. 10.1016/S0140-6736(21)01541-5 34883054

[4] Chen Y-L Chen WJ Lin K-C Shen L-J Gau SS-F. Prevalence of DSM-5 mental disorders in a nationally representative sample of children in Taiwan: methodology and main findings. Epidemiol Psychiatr Sci. (2019) 29:e15. 10.1017/S2045796018000793 30696515 PMC8061245

[5] Sheldrick RC Carter AS. State-level trends in the prevalence of autism spectrum disorder (ASD) from 2000 to 2012: a reanalysis of findings from the autism and developmental disabilities network. J Autism Dev Disord. (2018) 48:3086–92. 10.1007/s10803-018-3568-z 29654453 PMC6082680

[6] Blaxill M Rogers T Nevison C. Autism tsunami: the impact of rising prevalence on the societal cost of autism in the United States. J Autism Dev Disord. (2021) 52:2627–43. 10.1007/s10803-021-05120-7 34278527 PMC9114071

[7] Baxter AJ Brugha T Erskine HE Scheurer RW Vos T Scott JG. The epidemiology and global burden of autism spectrum disorders. Psychol Med. (2015) 45:601–13. 10.1017/S003329171400172X 25108395

[8] Papadopoulos D. Mothers' experiences and challenges raising a child with autism spectrum disorder: A qualitative study. Brain Sci. (2021) 11:309. 10.3390/brainsci11030309 33801233 PMC8001702

[9] Eldevik S Hastings RP Hughes JC Jahr E Eikeseth S Cross S. Meta-analysis of early intensive behavioral intervention for children with autism. J Clin Child Adolesc Psychol. (2009) 38:439–50. 10.1080/15374410902851739 19437303

[10] Reichow B Barton EE Boyd BA Hume K. Early intensive behavioral intervention (EIBI) for young children with autism spectrum disorders (ASD). Cochrane Database Syst Rev. (2012) 10:CD009260. 10.1002/14651858.CD009260.pub2 23076956

[11] Tiede G Walton KM. Meta-analysis of naturalistic developmental behavioral interventions for young children with autism spectrum disorder. Autism. (2019) 23:2080–95. 10.1177/1362361319836371 31018655

[12] Ministry of Health and Welfare in Taiwan. Number of Children with Developmental Problems for Early Intervention Services. (2021). Available online at: https://www.mohw.gov.tw/dl-22262-b0439eab-2785-467c-8ad4-d7b83c211914.html (accessed March 11, 2022).

[13] Huang P-H. The development and current situation of the early intervention for children with developmental delay in Taiwan. Int J Child Care Educ Policy. (2007) 1:45–58. 10.1007/2288-6729-1-1-45

[14] Chu C-L Chiang C-H Wu C-C Hou Y-M Liu J-H. Service system and cognitive outcomes for young children with autism spectrum disorders in a rural area of Taiwan. Autism. (2017) 21:581–91. 10.1177/1362361316664867 28610539

[15] Kaminski JW Valle LA Filene JH Boyle CL. A meta-analytic review of components associated with parent training program effectiveness. J Abnorm Child Psychol. (2008) 36:567–89. 10.1007/s10802-007-9201-9 18205039

[16] Oono IP Honey EJ McConachie H. Parent-mediated early intervention for young children with autism spectrum disorders (ASD). Evid Based Child Health. (2013) 8:2380–479. 10.1002/ebch.1952 23633377 PMC11831248

[17] Postorino V Sharp WG McCracken CE Bearss K Burrell TL Evans AN. A systematic review and meta-analysis of parent training for disruptive behavior in children with autism spectrum disorder. Clin Child Fam Psychol Rev. (2017) 20:391–402. 10.1007/s10567-017-0237-2 28600643

[18] Nevill RE Lecavalier L Stratis EA. Meta-analysis of parent-mediated interventions for young children with autism spectrum disorder. Autism. (2018) 22:84–98. 10.1177/1362361316677838 29490483

[19] Lovaas OI Koegel R Simmons JQ Long JS. Some generalization and follow-up measures on autistic children in behavior therapy. J Appl Behav Anal. (1973) 6:131–65. 10.1901/jaba.1973.6-131 16795385 PMC1310815

[20] Reichow B Kogan C Barbui C Smith I Yasamy MT Servili C. Parent skills training for parents of children or adults with developmental disorders: systematic review and meta-analysis protocol. BMJ Open. (2014) 4:e005799. 10.1136/bmjopen-2014-005799 25164537 PMC4156800

[21] Matson ML Mahan S Matson JL. Parent training: a review of methods for children with autism spectrum disorders. Res Autism Spectr Disord. (2009) 3:868–75. 10.1016/j.rasd.2009.02.003

[22] Lichtlé J Downes N Engelberg A Cappe E. The effects of parent training programs on the quality of life and stress levels of parents raising a child with autism spectrum disorder: A systematic review of the literature. Rev J Autism Dev. (2019) 7, 242–62. 10.1007/s40489-019-00190-x

[23] McConachie H Diggle T. Parent implemented early intervention for young children with autism spectrum disorder: a systematic review. J Eval Clin Pract. (2007) 13:120–9. 10.1111/j.1365-2753.2006.00674.x 17286734

[24] Aldred C Green J Emsley R McConachie H. Brief report: Mediation of treatment effect in a communication intervention for pre-school children with autism. J Autism Dev Disord. (2012) 42:447–54. 10.1007/s10803-011-1248-3 21512834

[25] Kasari C Gulsrud A Paparella T Hellemann G Berry K. Randomized comparative efficacy study of parent-mediated interventions for toddlers with autism. J Consult Clin Psychol. (2015) 83:554. 10.1037/a0039080 25822242 PMC4755315

[26] Bearss K Johnson C Smith T Lecavalier L Swiezy N Aman M. Effect of parent training vs parent education on behavioral problems in children with autism spectrum disorder: a randomized clinical trial. JAMA. (2015) 313:1524–33. 10.1001/jama.2015.3150 25898050 PMC9078140

[27] World Health Organization. WHO Caregivers Skills Training for Families of Children with Developmental Delays and Disorders. World Health Organization (n.d.). Available from: https://www.who.int/teams/mental-health-and-substance-use/treatment-care/whocaregivers-skills-training-for-families-of-children-with-developmental-delaysand-disorders (accessed March 11, 2022).

[28] World Health Organization. mhGAP Intervention Guide for Mental, Neurological and Substance Use Disorders in Non-Specialized Health Settings: Mental Health Gap Action Programme (mhGAP). (2016). Geneva, Switzerland: World Health Organization. 27786430

[29] Salomone E Pacione L Shire S Brown FL Reichow B Servili C. Development of the WHO caregiver skills training program for developmental disorders or delays. Front Psychiatry. (2019) 10:769. 10.3389/fpsyt.2019.00769 31780960 PMC6859468

[30] Tekola B Girma F Kinfe M Abdurahman R Tesfaye M Yenus Z. Adapting and pre-testing the World Health Organization's Caregiver Skills Training programme for autism and other developmental disorders in a very low-resource setting: findings from Ethiopia. Autism. (2020) 24:51–63. 10.1177/1362361319848532 31094208 PMC6927066

[31] Sengupta K Shah H Ghosh S Sanghvi D Mahadik S Dani A. World Health Organisation-Caregiver Skills Training (WHO-CST) Program: feasibility of delivery by non-specialist providers in real-world urban settings in India. J Autism Dev Disord. (2021) 1–18. 10.1007/s10803-021-05367-0 34853959

[32] Salomone E Settanni M McConachie H Suma K Ferrara F Foletti G. Pilot randomized controlled trial of the WHO caregiver skills training in public health services in Italy. J Autism Dev Disord. (2021):1-15. 10.1007/s10803-021-05297-x PMC9508213 34677755

[33] Salomone E Ferrante C Salandin A Ferrara F Torchio E Foletti G. Acceptability and feasibility of the World Health Organization's caregiver skills training implemented in the italian National Health System. Autism. (2022) 26:859–74. 10.1177/13623613211035228 34362266

[34] Singh NN Curtis WJ Ellis CR Nicholson MW Villani TM Wechsler HA. Psychometric analysis of the family empowerment scale. J Emot Behav Disord. (1995) 3:85–91. 10.1177/10634266950030020333268218

[35] Geier DA Kern JK Geier MR. A Comparison of the Autism Treatment Evaluation Checklist (ATEC) and the Childhood Autism Rating Scale (CARS) for the Quantitative Evaluation of Autism. J Ment Health Res Intellect Disabil. (2013) 6:255–67. 10.1080/19315864.2012.681340 23914277 PMC3725669

[36] Magiati I Moss J Yates R Charman T Howlin P. Is the autism treatment evaluation checklist a useful tool for monitoring progress in children with autism spectrum disorders? J Intellect Disabil Res. (2011) 55:302–12. 10.1111/j.1365-2788.2010.01359.x 21199043

[37] Wang S-C. A research of social support, empowerment and quality of life of parents with developmental delay children who have accepted early intervention services in Taipei City and New Taipei City. Taiwan: National Taiwan Normal University (2012).

[38] Little RJ Rubin DB. The analysis of social science data with missing values. Sociol Methods Res. (1989) 18:292–326. 10.1177/0049124189018002004

[39] White IR Royston P Wood AM. Multiple imputation using chained equations: issues and guidance for practice. Stat Med. (2011) 30:377–99. 10.1002/sim.4067 21225900

[40] Jennings MA Cribbie RA. Comparing pre-post change across groups: Guidelines for choosing between difference scores, ANCOVA, and residual change scores. J Data Sci. (2016) 14:205–29. 10.6339/JDS.201604_14(2).0002

[41] Mahapatra S Khokhlovich E Martinez S Kannel B Edelson SM Vyshedskiy A. Longitudinal epidemiological study of autism subgroups using Autism Treatment Evaluation Checklist (ATEC) Score. J Autism Dev Disord. (2020) 50:1497–508. 10.1007/s10803-018-3699-2 30062397 PMC7211200

[42] Kraiger K Ford JK Salas E. Application of cognitive, skill-based, and affective theories of learning outcomes to new methods of training evaluation. J Appl Psychol. (1993) 78:311–28. 10.1037/0021-9010.78.2.311

[43] Geiger KB LeBlanc LA Hubik K Jenkins SR Carr JE. Live training versus e-learning to teach implementation of listener response programs. J Appl Behav Anal. (2018) 51:220–35. 10.1002/jaba.444 29489011

[44] Kohrt BA Jordans MJD Rai S Shrestha P Luitel NP Ramaiya MK. Therapist competence in global mental health: Development of the ENhancing Assessment of Common Therapeutic factors (ENACT) rating scale. Behav Res Ther. (2015) 69:11–21. 10.1016/j.brat.2015.03.009 25847276 PMC4686771

[45] Dunst CJ Trivette CM. Let's be PALS: An evidence-based approach to professional development. Infants Young Children. (2009) 22:164–76. 10.1097/IYC.0b013e3181abe169

[46] Friedman M Woods J Salisbury C. Caregiver coaching strategies for early intervention providers: moving toward operational definitions. Infants Young Children. (2012) 25:62–82. 10.1097/IYC.0b013e31823d8f12

[47] Chao K-Y Chang H-L Chin W-C Li H-M Chen S-H. How Taiwanese parents of children with autism spectrum disorder experience the process of obtaining a diagnosis: a descriptive phenomenological analysis. Autism. (2018) 22:388–400. 10.1177/1362361316680915 28205453

[48] Rivard M Morin M Mercier C Terroux A Mello C Lépine A. Social validity of a training and coaching program for parents of children with autism spectrum disorder on a waiting list for early behavioral intervention. J Child Fam Stud. (2017) 26:877–87. 10.1007/s10826-016-0604-5

[49] Mahapatra S Vyshedsky D Martinez S Kannel B Braverman J Edelson SM. Autism Treatment Evaluation Checklist (ATEC) Norms: A “Growth Chart” for ATEC Score Changes as a Function of Age. Children. (2018) 5:25. 10.3390/children5020025 29462954 PMC5835994

[50] Trembath D Gurm M Scheerer NE Trevisan DA Paynter J Bohadana G. Systematic review of factors that may influence the outcomes and generalizability of parent-mediated interventions for young children with autism spectrum disorder. Autism Res. (2019) 12:1304–21. 10.1002/aur.2168 31294532

