# Supplementary material for: Promotion and implementation effectiveness of World Health Organization's Caregiver Skills Training program in Taiwan
Source: Front Psychiatry. 2022 Aug 31;13:904380. doi: 10.3389/fpsyt.2022.904380 (PMC9470946; doi:10.3389/fpsyt.2022.904380)
Supplement: Supplementary file 1 [file Data_Sheet_1.docx]

**Supplementary Table 1. Differences between followed and non-followed families**

1. Caregivers

|  | Mean (SD) | |  |
| --- | --- | --- | --- |
|  | Followed (n=50) | Non-followed (n=44) | Statistics |
| Child age, year [mean, (SD)] | 3.98 (1.11) | 3.71 (1.02) | t =−1.21, *p*=0.228 |
| Child sex (Male: Female) | 38:12 | 34:10 | x^2^=0.02, *p*=0.884 |
| Treated: non-treated | 27:23 | 21:23 | x^2^=0.37, *p*=0.554 |
| Baseline autistic symptoms [mean, (SD)] | 59.15 (23.22) | 64.48 (27.99)^a^ | t =0.99, *p*=0.323 |
| Caregiver age, year [mean, (SD)] | 37.96 (6.12) | 39.25 (5.42) | t =1.08, *p*=0.285 |
| Caregiver sex (Male: Female) | 4:46 | 3:41 | t =0.05, *p*=0.828 |
| Caregiver educational levels  (Junior/High/College/Graduate) | 1/9/29/11 | 2/5/26/11 | x^2^=1.26, *p*=0.738 |
| Baseline caregiver knowledge [mean, (SD)] | 95.77 (8.38) | 96.40 (7.66)^b^ | t =0.38, *p*=0.709 |
| Baseline caregiver confidence [mean, (SD)] | 39.22 (7.81) | 38.21 (8.36)^b^ | t =−0.60, *p*=0.549 |
| Baseline family empowerment [mean, (SD)] | 125.92 (14.49) | 125.39 (15.59)^b^ | t =0.47, *p*=0.641 |

1. Children

|  | Mean (SD) | |  |
| --- | --- | --- | --- |
|  | Followed (n=50) | Non-followed (n=41) | Statistics |
| Child age, year [mean, (SD)] | 3.98 (1.11) | 3.69 (1.02) | t =−1.30, *p*=0.196 |
| Child sex (Male: Female) | 38:12 | 32:9 | x^2^=0.05, *p*=0.817 |
| Treated: non-treated | 27:23 | 20:21 | x^2^=0.25, *p*=0.620 |
| Baseline autistic symptoms [mean, (SD)] | 59.15 (23.22) | 66.04 (27.60)^c^ | t =1.27, *p*=0.207 |
| Caregiver age, year [mean, (SD)] | 37.96 (6.12) | 38.85 (5.23) | t =0.74, *p*=0.462 |
| Caregiver sex (Male: Female) | 4:46 | 2:39 | x^2^=0.36, *p*=0.550 |
| Caregiver educational levels  (Junior/High/College/Graduate) | 1/9/29/11 | 2/5/24/10 | x^2^=1.12, *p*=0.773 |
| Baseline caregiver knowledge [mean, (SD)] | 95.77 (8.38) | 96.19 (7.65)^d^ | t =0.25, *p*=0.803 |
| Baseline caregiver confidence [mean, (SD)] | 39.22 (7.81) | 38.04 (8.53)^d^ | t =−0.69, *p*=0.494 |
| Baseline family empowerment [mean, (SD)] | 125.92 (14.49) | 125.46 (15.92)^d^ | t =0.48, *p*=0.633 |

Abbreviations: SD, standard deviation.

Note: Autistic symptoms were assessed by Autism Treatment Evaluation Checklist. ^a^ Only 41 children had this data; ^b^ Only 42 caregivers completed these questionnaires. ^c^ Only 38 children had this data; ^d^ Only 40 caregivers completed these questionnaires.

**Supplementary Table 2. Differences among CST groups delivered by facilitators with different experience levels.**

1. Caregivers

|  | Mean (SD) | | |  |
| --- | --- | --- | --- | --- |
|  | A (n=21) | B (n=30) | C (n=43) | Statistics |
| Child age, year [mean, (SD)] | 3.63 (0.80) | 3.70 (1.08) | 4.07 (1.16) | F=1.73, *p*=0.188 |
| Child sex (Male: Female) | 17:4 | 23:7 | 32:11 | x^2^=0.34, *p*=0.845 |
| Treated: non-treated | 11:10 | 9:21 | 26:17 | x^2^=6.70, *p*=0.035 |
| Baseline autistic symptoms severity [mean, (SD)] | 56.67 (24.34) | 59.81 (23.77)^a^ | 65.16 (27.12)^b^ | F=0.85, *p*=0.434 |
| Caregiver age, year [mean, (SD)] | 37.43 (6.32) | 39.80 (5.54) | 38.26 (5.71) | F=1.13, *p*=0.333 |
| Caregiver sex (Male: Female) | 0:21 | 1:29 | 6:37 | x^2^=5.07, *p*=0.079 |
| Caregiver educational levels  (Junior/High/College/Graduate) | 1/3/15/2 | 0/3/19/8 | 2/8/21/12 | x^2^=5.94, *p*=0.430 |
| Baseline caregiver knowledge [mean, (SD)] | 94.57 (9.55) | 97.50 (7.90)^c^ | 95.80 (7.28) | F=0.75, *p*=0.478 |
| Baseline caregiver confidence [mean, (SD)] | 38.05 (11.12) | 39.30 (7.70)^c^ | 38.74 (6.48) | F=0.11, *p*=0.897 |
| Baseline family empowerment [mean, (SD)] | 122.97 (19.59) | 125.48 (10.25)^c^ | 124.79 (15.31) | F=0.15, *p*=0.864 |

1. Children

|  | Mean (SD) | | |  |
| --- | --- | --- | --- | --- |
|  | A (n=21) | B (n=30) | C (n=40) | Statistics |
| Child age, year [mean, (SD)] | 3.63 (0.80) | 3.70 (1.08) | 4.07 (1.17) | F=1.64, *p*=0.203 |
| Child sex (Male: Female) | 17:4 | 23:7 | 30:10 | x^2^=0.28, *p*=0.871 |
| Treated: non-treated | 11:10 | 9:21 | 24:16 | x^2^=6.36, *p*=0.042 |
| Baseline autistic symptoms severity [mean, (SD)] | 56.67 (24.34) | 59.81 (23.77)^d^ | 66.72 (26.62)^e^ | F=1.22, *p*=0.305 |
| Caregiver age, year [mean, (SD)] | 37.43 (6.32) | 39.80 (5.54) | 37.78 (5.46) | F=1.45, *p*=0.245 |
| Caregiver sex (Male: Female) | 0:21 | 2:28 | 5:35 | x^2^=3.10, *p*=0.213 |
| Caregiver educational levels  (Junior/High/College/Graduate) | 1/3/15/2 | 0/3/19/8 | 2/8/19/11 | x^2^=6.35, *p*=0.386 |
| Baseline caregiver knowledge [mean, (SD)] | 94.57 (9.55) | 97.50 (7.90)^f^ | 95.57 (7.23) | F=0.82, *p*=0.447 |
| Baseline caregiver confidence [mean, (SD)] | 38.05 (11.12) | 39.30 (7.70)^f^ | 38.60 (6.61) | F=0.13, *p*=0.882 |
| Baseline family empowerment [mean, (SD)] | 122.97 (19.59) | 125.48 (10.25)^f^ | 124.83 (15.64) | F=0.15, *p*=0.865 |

Abbreviations: SD, standard deviation.

Note: Group A refers to the CST group delivered by 2 experienced facilitators. Group B refers to the CST group delivered jointly by 1 new facilitator and 1 experienced facilitator. Group C refers to the CST group delivered by 2 new facilitators. Baseline autistic symptoms were assessed by the total score of Autism Treatment Evaluation Checklist. ^a^ Only 28 children had this data; ^b^ Only 42 children had this data; ^c^ Only 29 caregivers completed these questionnaires. ^d^ Only 28 children had this data; ^e^ Only 39 children had this data; ^f^ Only 29 caregivers completed these questionnaires; There were total 18 caregiver groups in the analysis (A: 4 groups; B: 6 groups; C: 8 groups).

**Supplementary Table 3. Differences of caregiver outcomes and child outcomes among CST groups delivered by facilitators with different experience levels.**

1. Caregivers

| Changes of caregiver outcomes | Mean (SD) | | | Statistic (F) | *p* value | Effect size (η^2^) | Post-Hoc |
| --- | --- | --- | --- | --- | --- | --- | --- |
|  | A  (n=21) | B  (n=28) | C  (n=38) |  |  |  |  |
| Knowledge | 6.48 (7.61) | 6.84 (7.40) | 7.16 (5.49) | 0.20 | 0.816 | 0.004 | − |
| Confidence | 9.71 (7.58) | 9.85 (8.20) | 10.97 (7.69) | 0.46 | 0.631 | 0.007 | − |
| Empowerment | 12.16 (8.78) | 8.78 (15.85) | 10.48 (9.70) | 1.32 | 0.273 | 0.027 | − |

1. Children

| Changes of child outcomes | Mean (SD) | | | Statistic (F) | *p* value | Effect size (η^2^) | Post-Hoc |
| --- | --- | --- | --- | --- | --- | --- | --- |
|  | A  (n=19) | B  (n=28) | C  (n=38) |  |  |  |  |
| Speech/language/communication | −2.04 (2.34) | −1.74 (3.89) | −2.73 (3.98) | 0.15 | 0.864 | 0.003 | − |
| Sociability | −1.27 (4.36) | −0.54 (5.73) | −2.27 (6.03) | 0.18 | 0.832 | 0.004 | − |
| Sensory/cognitive awareness | −1.01 (3.10) | −1.95 (5.26) | −2.54 (5.31) | 0.58 | 0.565 | 0.013 | − |
| Health/physical behaviors | −1.94 (8.94) | −0.80 (7.15) | −2.82 (8.83) | 0.75 | 0.477 | 0.016 | − |
| Total | −6.26 (11.94) | −5.02 (15.81) | −10.36 (19.62) | 0.56 | 0.575 | 0.013 | − |

Abbreviations: SD, standard deviation.

Note: Group A refers to the CST group delivered by 2 experienced facilitators. Group B refers to the CST group delivered jointly by 1 new facilitator and 1 experienced facilitator. Group C refers to the CST group delivered by 2 new facilitators.
